# Effects of clothianidin on aquatic communities: Evaluating the impacts of lethal and sublethal exposure to neonicotinoids

**DOI:** 10.1371/journal.pone.0174171

**Published:** 2017-03-23

**Authors:** Jesse C. Miles, Jessica Hua, Maria S. Sepulveda, Christian H. Krupke, Jason T. Hoverman

**Affiliations:** 1 Department of Forestry and Natural Resources, Purdue University, West Lafayette, Indiana, United States of America; 2 Biological Sciences Department, Binghamton University (SUNY), Binghamton, New York, United States of America; 3 Department of Entomology, Purdue University, West Lafayette, Indiana, United States of America; Institut Sophia Agrobiotech, FRANCE

## Abstract

The widespread usage of neonicotinoid insecticides has sparked concern over their effects on non-target organisms. While research has largely focused on terrestrial systems, the low soil binding and high water solubility of neonicotinoids, paired with their extensive use on the landscape, puts aquatic environments at high risk for contamination via runoff events. We assessed the potential threat of these compounds to wetland communities using a combination of field surveys and experimental exposures including concentrations that are representative of what invertebrates experience in the field. In laboratory toxicity experiments, LC_50_ values ranged from 0.002 ppm to 1.2 ppm for aquatic invertebrates exposed to clothianidin. However, freshwater snails and amphibian larvae showed high tolerance to the chemical with no mortality observed at the highest dissolvable concentration of the insecticide. We also observed behavioral effects of clothianidin. Water bugs, *Belostoma flumineum*, displayed a dose-dependent reduction in feeding rate following exposure to clothianidin. Similarly, crayfish, *Orconectes propinquus*, exhibited reduced responsiveness to stimulus with increasing clothianidin concentration. Using a semi-natural mesocosm experiment, we manipulated clothianidin concentration (0.6, 5, and 352 ppb) and the presence of predatory invertebrates to explore community-level effects. We observed high invertebrate predator mortality with increases in clothianidin concentration. With increased predator mortality, prey survival increased by 50% at the highest clothianidin concentration. Thus, clothianidin contamination can result in a top-down trophic cascade in a community dominated by invertebrate predators. In our Indiana field study, we detected clothianidin (max = 176 ppb), imidacloprid (max = 141 ppb), and acetamiprid (max = 7 ppb) in soil samples. In water samples, we detected clothianidin (max = 0.67 ppb), imidacloprid (max = 0.18 ppb), and thiamethoxam (max = 2,568 ppb). Neonicotinoids were detected in >56% of soil samples and >90% of the water samples, which reflects a growing understanding that neonicotinoids are ubiquitous environmental contaminants. Collectively, our results underscore the need for additional research into the effects of neonicotinoids on aquatic communities and ecosystems.

## Introduction

Neonicotinoid insecticides, which account for 26% of the global insecticide market, have recently become the most widely used insecticide class worldwide [1]. Developed in the 1980s, neonicotinoids first came into regular use with imidacloprid starting in the early 1990s. Since that time additional active ingredients have been developed and classified into three groups: *N-*nitroguanidines (imidacloprid, thiamethoxam, clothianidin, dinotefuran), nitromethylenes (nitenpyram), and *N*-cyanoamidines (acetamiprid and thiacloprid) [2]. Currently, thiamethoxam and its breakdown product clothianidin dominate usage in North American cropping systems [3]. The increasing usage of neonicotinoids has been fueled by their relatively low toxicity to vertebrate species [4]. Neonicotinoids target the post-synaptic nicotinic acetylcholine receptor, causing paralysis and death. Because neonicotinoids bind more strongly to insect receptors than vertebrate receptors and invertebrates have a higher ratio of nicotinic receptors, they generally have low toxicity to vertebrate species [4]. A key driver of rapid neonicotinoid adoption in North America is the ability to apply them prophylactically as a seed dressing to some of the most widely grown annual crops [3]. As seeds germinate, the insecticide is incorporated into the plant and distributed systemically during growth. This process is facilitated by the high water solubility of neonicotinoids [4]. Although neonicotinoids can be used as spray applications, approximately 60% of applications are as seed dressings [2]. The prophylactic application of neonicotinoids to virtually all seeds of corn, soybeans and other annual crops without prior knowledge of the season’s pest populations has raised concern over the environmental risks associated with their use [3].

Only a small fraction of neonicotinoid active ingredient applied to seeds is taken up by plants. For example, in a container study, less than 20% of the imidacloprid applied to corn seeds was later found in the plant, the remainder presumably retained in soils and water [5]. These findings raise questions about environmental fate, as neonicotinoids generally have exceptionally high water solubility values; clothianidin and thiamethoxam, the two compounds used most frequently in our study area in the Midwestern US [3], have solubility values of 0.327 g L^-1^ and 4.1 g L^-1^, respectively [6,7]. While the high water solubility and low soil binding by neonicotinoids facilitates translocation by plants, it can lead to significant leaching into ground water, streams, and ponds. For example, imidacloprid was detected in 89% surface water samples (n = 75) in California [8]. Similarly, the Washington State Departments of Agriculture and Ecology have detected imidacloprid (max = 0.705 ppb, mean = 0.06 ppb) during monitoring studies of salmon-bearing rivers and streams [9]. Additionally, at least one of four different neonicotinoid compounds (clothianidin, thiamethoxam, acetamiprid, imidacloprid) were found in 16 to 91% of water and sediment samples in the Canadian Prairie Pothole Region, dependent on time of sampling [10]. In a review of 29 studies from nine countries, neonicotinoids were common contaminants of surface waters [11].

Given the frequency of detection of neonicotinoids in aquatic systems, many recent studies have explored the potential lethal and sublethal effects of neonicotinoids on aquatic species (reviewed in [12,13]). Aquatic insects are generally more sensitive to neonicotinoids compared to other aquatic species (e.g., mollusks, crustaceans, fish), which is not surprising given their mode of action [13]. In addition to their effects on mortality, neonicotinoids have been shown to reduce feeding rates, movement, fecundity, developmental rates, and growth in aquatic insects [14–21]. However, the majority of this research has focused on imidacloprid, which was the first widely applied neonicotinoid and is rarely used in modern row crop agriculture production systems. There is a dearth of information on the toxicological effects of the neonicotinoids that are most commonly used presently, including thiamethoxam and its metabolite clothianidin [13].

While laboratory experiments can provide a wealth of information on the effects of pesticides on individuals and populations, community-level experiments can broaden our perspective of how natural systems are likely to respond to these common stressors, including neonicotinoids [22–27]. Ecological communities are complex systems composed of species representing different trophic levels and functional groups that directly and indirectly interact. Direct interactions including competition, predation, and parasitism have routinely been explored in ecological research [28]. Moreover, there is increasing focus on how these direct interactions can indirectly influence other species within communities (e.g., trophic cascades; [29]). Indeed, indirect interactions within communities are mediated by a combination of changes in species abundance and changes in species traits (e.g., behavior). Because neonicotinoids are designed to target insects, they should have predictable direct effects (e.g., mortality) and more difficult to quantify sublethal effects (e.g., reduced foraging and activity) on predatory invertebrates [23,30]. Broadly, macroinvertebrates represent a significant component of the biodiversity in many freshwater water systems (e.g., ponds, wetlands, streams; [31]). Moreover, insects are a dominant predatory guild in lentic systems that lack fish [32]. Using basic food web theory, we would predict that the elimination of predatory insects or reductions in their foraging activity in a system will lead to a “top-down” effect that indirectly increases the abundance of prey species [33–35]. By integrating research across multiple ecological scales (e.g., individuals, populations, communities), we can develop a broader understanding of how neonicotinoids can influence community structure and function.

We combined laboratory and mesocosm experiments with field surveys to assess the potential effects of neonicotinoids on wetland species. Our experiments focused on the neonicotinoid clothianidin, which is a breakdown product of the widely used neonicotinoid thiamethoxam but also used as an active ingredient. In fact, within the last decade, clothianidin has become the dominant neonicotinoid used in North America for many applications. It is registered for use as a foliar insecticide and as a seed treatment for most annual crops [2,3]. Given the shift from imidacloprid to thiamethoxam and clothianidin as the dominant neonicotinoid active ingredients used in agriculture, there is a need to evaluate the risk that these compounds pose to natural systems. To date, clothianidin toxicity testing for aquatic species has been limited to a small number of aquatic invertebrates (e.g., *Chironomus riparius*, *Mysidopsis bahia*, *Daphnia*), with LC_50_ estimates ranging from 0.022 ppm to 119 ppm [36,37]. Given the broad diversity of species, particularly invertebrates, that inhabit aquatic systems, there is a need for studies that expand beyond traditional model species. Moreover, the sublethal effects of clothianidin on aquatic taxa and the community-level implications of typical exposures are largely unknown. In order to provide a baseline for further work in aquatic systems, our experimental objectives were to assess the lethal and sublethal effects of clothianidin to common wetland invertebrate (e.g., snails, insects, crustaceans) and vertebrate (i.e. amphibian) species in the Upper Midwestern United States (Indiana), where the use of clothianidin and thiamethoxam is as intensive as any region in the country [38]. To assess lethal effects, we conducted toxicity assays (i.e. 48 h LC_50_ tests). Additionally, we examined the sublethal effects of clothianidin exposure on movement and foraging activity (i.e. predation rates). Building upon results of our laboratory experiments, we conducted a mesocosm experiment to examine the effects of clothianidin on aquatic communities with different trophic structures (i.e. presence or absence of invertebrate predators). Finally, we used a field survey to collect weekly soil and water samples across multiple sites in central Indiana to determine the presence and environmental range of neonicotinoids on the landscape.

## Methods

### LC_50_ tests

We examined the toxicity of the neonicotinoid clothianidin to 10 aquatic macroinvertebrates and three larval anuran species using 48 hr LC_50_ (lethal concentration to 50% of organisms exposed) tests. The scale of our tests and volumes of water required precluded us from using technical grade active ingredient due to cost, and we used a formulated product, Arena 0.25% granules (Valent Corp., Walnut Creek, CA), to formulate our concentration regimes. Given that we used a commercial formulation of clothianidin, we cannot separate effects of the active ingredient from those of inert ingredients. Information on each species, including number of individuals used, is included in Table 1. All species were collected from ponds located near the Purdue Wildlife Area (PWA), Aquatic Research Lab, and Martell Forest in West Lafayette, IN U.S.A. between May and July of 2014 and 2015. After collection, the species were housed indoors at the PWA-Animal Care Facility under a 14:10-h light:dark cycle for no longer than 48 h prior to experimental use. Animals were housed individually in 1-L plastic containers filled with 0.5 L of UV-sterilized, filtered well water.

**Table 1:** Species and their respective experimental units and dosage concentrations. A single individual was assigned to each replicate.

Species	Order	Trophic position	Container Volume (mL)	Replicates	Nominal concentrations (ppm)
*Graphoderus fascicollis*	Coleoptera	Predator	10	10	0, 0.001, 0.010, 0.25, 0.50, 0.100
*Anax junius*	Odonata	Predator	500	4	0, 0.5, 1, 5, 10, 20
*Lestes unguiculatus*	Odonata	Predator	10	10	0, 0.5, 1, 3, 5, 10
*Plathemis lydia*	Odonata	Predator	500	10	0, 0.05, 0.5, 1, 10, 50
*Belostoma flumineum*	Hemiptera	Predator	110	8	0, 0.010, 0.050, 0.100, .250, 0.500
*Hesperocorixa atopodonta*	Hemiptera	Herbivore	100	10	0, 0.01, 0.025, 0.05, 0.1, 0.3
*Notonecta undulata*	Hemiptera	Predator	100	10	0, 0.01, 0.025, 0.05, 0.1, 0.3
*Orconectes propinquus*	Decapoda	Predator	1000	10	0, 0.5, 0.15, 0.3, 0.5, 0.6, 0.9, 1, 5, 20
*Physa acuta*	Pulmonata	Herbivore	500	5	0, 327
*Helisoma trivolvis*	Pulmonata	Herbivore	500	5	0, 327
*Hyla versicolor*	Anura	Herbivore	500	5	0, 327
*Lithobates clamitans*	Anura	Herbivore	500	5	0, 327
*Lithobates pipiens*	Anura	Herbivore	500	5	0, 327

We conducted individual LC_50_ tests for each species. Because the species differed in body size, we varied the size of our experimental units (10–1000 mL glass containers; Table 1). A single individual was placed into each experimental unit for the tests. Because little was known regarding the toxicity of clothianidin, we first conducted range-finding studies to determine lethal concentrations for each species. Based on these studies, we selected 6 to 10 nominal concentrations for each species and each concentration was replicated 4 to 15 times based on the availability of organisms (Table 1). In accordance with standard toxicity protocols, we did not feed individuals during the 48-h tests [39]. Tests were conducted under a 14:10-h light:dark cycle.

We prepared a stock solution of 300 ppm clothianidin using Arena 0.25% granules mixed with filtered, ultraviolet-irradiated well water. The solution was filtered using Whatman GF/C filters (90 mm) and stored in glass amber jugs for no more than 1 h before addition to the experimental units. To achieve the desired nominal concentrations, we used micropipettes to add stock solution to each container. Due to the small volume used in the experiments for the damselfly nymphs and beetle larvae, we premixed concentrations using a serial dilution for increased accuracy. We stirred the water in each experimental unit prior to the addition of the animals. To quantify the insecticide concentration, a mock stock solution was prepared in a glass amber jar and immediately taken for chemical analysis to determine preparation accuracy. The experimental units were monitored for mortality every 4 h for 48 h. We performed a probit analysis using SPSS software to determine LC_50_ values and 95% confidence intervals.

### Sublethal experiments with tadpoles

We conducted a laboratory experiment to explore the potential sublethal effects of clothianidin exposure on tadpole behavior (i.e. activity). The focal species was the northern leopard frog, *Lithobates pipiens*. The experiment consisted of a no-insecticide control or exposure to three concentrations of clothianidin (0.25 ppm, 0.5 ppm, or 1 ppm). All stock solutions for the experiments were prepared as described for the LC_50_ tests. Each treatment was replicated five times for a total of 20 experimental units. Our experimental units were 10-L plastic tubs filled with 2 L of UV-sterilized, filtered well water. We added 10 tadpoles to each experimental unit following the addition of the insecticide. Our behavioral observations were conducted by scan sampling [40]. For each tub, we recorded the number of individuals that were active (e.g., tail movement, movement through the water column). We conducted observations 30 min post-dosage, 1 h post-dosage, and then every 12 h for 48 h. For each set of observations, we conducted five scan samples for each tub and calculated the mean activity as our response variable. We used repeated-measures analysis of variance (ANOVA) to assess treatment effects over time using SPSS. We conducted mean comparisons using Bonferroni correction.

### Sublethal experiments with predators

We conducted laboratory experiments to explore the sublethal effects of clothianidin exposure on predator behavior (i.e. response to stimulus) and predator-prey interactions (i.e. predation rates). The focal predator species in these experiments were crayfish, *Orconectes propinquus*, and water bugs, *Belostoma flumineum*. All stock solutions for the experiments were prepared as described for the LC_50_ tests.

The feeding rate experiments consisted of a no-insecticide control or exposure to three concentrations of clothianidin. For crayfish, the three insecticide concentrations were 0.05 ppm, 0.1 ppm, and 0.2 ppm while the three concentrations for water bugs were 0.01 ppm, 0.05 ppm, and 0.1 ppm. The experimental units were 10-L tubs filled with 2 L of UV-sterilized, filtered well water. We added 10 snails (*Physa acuta*) and introduced a single predator to each experimental unit. Clothianidin was added to the tubs immediately prior to predator addition. The water was stirred to equally distribute the insecticide. We replicated each treatment six times for the water bug experiment and 10 times for the crayfish experiment, resulting in 24 and 40 total units, respectively. We checked twice daily for the number of snails consumed and removed dead snails from the tubs. The experiment was terminated after 4 d for the water bugs and 8 d for the crayfish. Our response was the total number of snails consumed in each experimental unit at the end of the experiment. We used ANOVA to assess the effects of clothianidin on prey consumption using SPSS. We conducted mean comparisons using Bonferroni correction.

We also examined the effects of clothianidin exposure on crayfish behavior. We used the same experimental design described above with the exception that the experimental units were 2-L container filled with 1 L of water. Stimuli were introduced by approaching experimental units, then touching the center of the cephalothorax using a disposable transfer pipette. A reaction was measured as either an escape movement away from stimulus, or aggressive stance towards the stimulus. This was performed 1 h post exposure, then every 24 h for 7 d (n = 8 total observations per individual). At the end of the experiment, we calculated the proportion of observations with responses to the stimulus as the response variable. We used ANOVA to assess the effect of clothianidin exposure on stimulus response. We conducted mean comparisons using Bonferroni correction.

### Mesocosm experiment

We investigated the potential interactive effects of clothianidin and predation on aquatic communities using a semi-natural mesocosm experiment. The herbivore trophic level consisted of amphibian larvae, freshwater snails, and zooplankton. The predator trophic level consisted of larval dragonflies (*Anax junius*), water bugs (*Belostoma flumineum*), backswimmers (*Notonecta undulata*), and crayfish (*Orchonectes propinquus*). Dragonflies, water bugs, and crayfish were selected because they will consume tadpoles and snails while backswimmers were selected because they will consume zooplankton. Based on previous research with imidacloprid [11,13,41], we expected the herbivores to be tolerant of clothianidin but the predatory insects and crayfish to be sensitive to it. Thus, we predicted that clothianidin exposure would have negative effects on predator survival and behavior, which would indirectly benefit herbivore survival and growth. Moreover, we expected sublethal effects on predator behavior to be the main driver of effects on herbivore responses at the low clothianidin concentration and lethal effects to dominate at the high clothianidin concentration.

The experiment was conducted at the PWA in July 2014. We used a complete randomized factorial design consisting of two predator treatments (presence or absence of invertebrate predators) crossed with three nominal concentrations of clothianidin (0, 10, or 500 ppb). The 10 ppb treatment was selected to reflect clothianidin concentrations that have been detected in water samples near agricultural fields [42] and expected to be sublethal to invertebrates. The 500 ppb treatment was selected to represent a worst-case scenario that would be potentially lethal to predatory invertebrates. We replicated the six treatments nine times for a total of 54 experimental units. Our experimental units were 1200-L cattle tanks located in an open field with no tree cover.

Between 17 and 19 June, we filled each tank with 595 L of well water and then covered the tank with 70% shade cloth to prevent unwanted colonization of insects and amphibians. On 22 June, we added 20 g of commercial rabbit chow (Small World Complete Rabbit Feed) and 200 g of dry leaf litter (primarily *Quercus* spp.) to provide an initial nutrient source and refuges, respectively. Additionally, we collected pond water from a local pond, removed all unwanted macroinvertebrates, and added a 500-mL sample from the mixture to each tank. This sample provided in initial source of algae (periphyton and phytoplankton) for the tanks. On 30 June, we placed two 10 x 10 cm clay tiles (oriented vertically and facing north) in each tank. After allowing seven days for algal populations to develop, we seeded each tank with a zooplankton assemblage gathered from previously established mesocosms at our facility.

We assembled aquatic communities that are common across wetlands in our region [43,44]. Our base community (no-predator treatments) consisted of two species of larval amphibians (northern leopard frogs, *Lithobates pipiens*, and green frogs, *L*. *clamitans*) and two species of freshwater snails (*Helisoma trivolvis* and *Physa acuta*). We collected eight egg masses of northern leopard frogs from a local pond and reared the hatchlings in 100-L culture pools filled with 70 L of well water covered with 70% shade cloth. Tadpoles were fed rabbit chow until used in the experiment. We collected green frog tadpoles from a nearby wetland on 4 July for use in the experiment. On 7 July, we added 20 northern leopard frog tadpoles and 10 green frog tadpoles to each tank. The snail species were also collected from local ponds between 30 June and 4 July. On 7 July, we added 30 individuals of each snail species to each tank. Our predator species consisted of water bugs (*B*. *flumineum*; n = 5), backswimmers (*N*. *undulate;* n = 5), dragonfly larvae (*A*. *junius;* n = 2), and crayfish (*O*. *propinquus;* n = 10) collected from local ponds and reared in the laboratory until used in the experiment. The densities of all species were within the range found in wetlands [43,44]. The predators were added to the tanks on 7 July after the addition of the prey species.

The tanks were dosed on 7 July with 18.4 and 921 mL of clothianidin stock solution (323 ppm) to achieve nominal concentrations of 10 and 500 ppb, respectively. The water in each tank was gently agitated with a metal rod to distribute the insecticide throughout the tank. A 200-mL sample was immediately collected from five randomly selected tanks in each treatment. The five samples were mixed together and a 200-mL sample of the pooled sample was removed for chemical analysis to determine the actual concentrations achieved in the treatments (S1 Table). At day 0, actual concentrations were 5 ppb and 352 ppb for the 10 and 500 ppb treatments, respectively. We also note that clothianidin was detected in our well water; the clothianidin concentration in our control tanks was 0.6 ppb. Given that the actual concentrations were less than our nominal concentrations, we will refer to the actual concentrations below. Additionally, we collected water samples on day 21 of the experiment to assess degradation of clothianidin over time; concentrations were 0.3, 1.5, and 77.6 ppb for the 0, 10, and 500 ppb treatments, respectively. A mock solution was also made to determine accuracy of stock solutions. All samples were stored in glass amber jars and analyzed within 24 h of collection at the Purdue University Bindley Bioscience Lab using a triple quadrupole (QQQ) liquid chromatography/mass spectrometer (LC/MS).

During the experiment, we measured pH, temperature, conductivity, periphyton biomass, phytoplankton (Chlorophyll *a*), and zooplankton abundance. Sampling methods and results are presented in S1 Appendix, S2 and S3 Tables, and S1 and S2 Figs. The experiment was taken down 21 d post insecticide exposure. Upon termination, we removed all of the amphibians, snails, and predators from the tanks. Individuals were euthanized and then preserved in 10% formalin (amphibians and snails) or 70% ethanol (predators). For each tank, we determined the number of surviving individuals for each species.

Predator mortality in our mesocosm experiment did not meet the assumptions of parametric analyses. Thus, we used a Kruskal-Wallis test to determine the effect of clothianidin on overall predator mortality and the mortality of each predator species. We used generalized linear models (GLM) to test for the effects of predators, clothianidin, and the predator*clothianidin interaction on overall prey mortality and the mortality of each prey species. For significant univariate effects, we conducted mean comparisons using Bonferroni correction.

### Field survey

**Fig 1:**
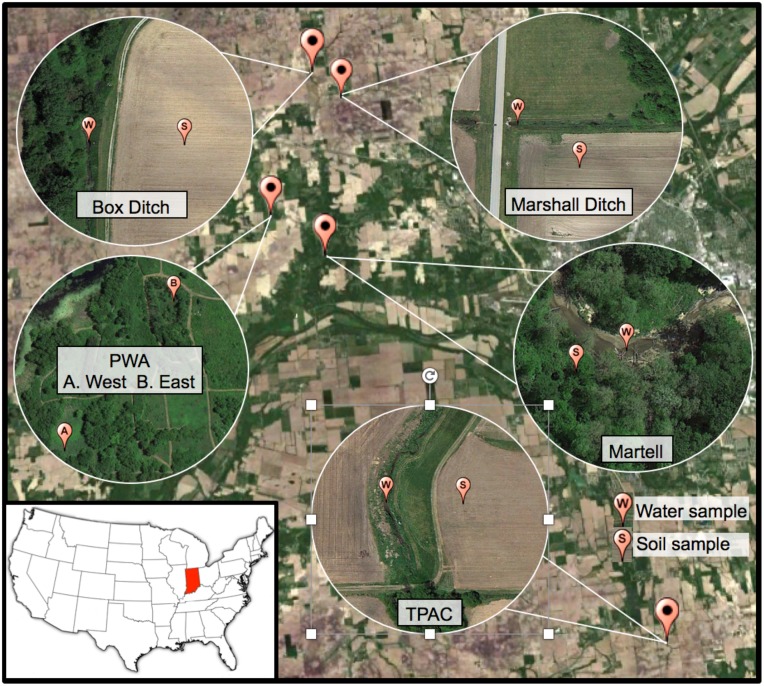
Map of field sites and sampling locations in Tippecanoe Co., Indiana, U.S.A. For each site, the location of water (W) and/or soil (S) samples is indicated. Our study sites were the Purdue Wildlife Area (PWA East Pond [40.452261°, -87.055185°] and PWA West Pond [40.450746°, -87.052397°]), Martell Forest (40.435215°, -87.029180°), Throck Morton Purdue Agricultural Center (TPAC, [40.295857°, -86.899099°]), and the Purdue Animal Farm (Box [40.503325°, -87.026892°] and Marshall [40.492395°, -87.014538°]).

We conducted field surveys to determine neonicotinoid concentrations in soil and water samples from multiple sites in Tippecanoe Co., Indiana (Fig 1). We tested for the most commonly used neonicotinoids in our area (acetamiprid, clothianidin, imidacloprid, and thiamethoxam). TPAC, Box, and Marshall were agricultural sites whereas Martell Forest served as a reference site. However, we note that Martell Forest is embedded within an agricultural landscape. Each of these four sites has an associated stream or ditch that served as a location for our water samples. The PWA was selected because it contains wetland areas that would allow us to assess neonicotinoid concentrations in lentic water bodies, including sites that served as sources for our experimental animals. We conducted soil and water sampling at Martell Forest, TPAC, Box, and Marshall. Sampling was performed at each site two weeks prior to planting and weekly from two through eight weeks post-planting. For the two sites at the PWA, we only conducted water sampling.

For water samples, we randomly collected 100-mL samples from three different locations. The three samples were pooled together into a 500-mL amber Nalgene bottle and frozen to prevent degradation of compounds until processing. Once thawed, we removed 3 replicate samples of 20 mL from each bottle for analysis. The samples were collected in amber vials to determine neonicotinoid concentrations. The samples were first mixed with 10 μL of a 1–10 ng/μL analytical grade standards, then poured through *Oasis Waters* SPE cartridges, with 3 mL of acetonitrile used to elute the sample prior to measurement. We then used QQQ LC/MS to determine neonicotinoid concentrations. For each sample, the reported analytical results are the mean of the three replicate measurements (see S1 Appendix for concentration determination).

Soil samples were collected from five randomly chosen locations in the fields near the water collection sites. Soil cores were taken with the top six inches of topsoil removed. The five samples were mixed together to form a single sample and held in opaque paper bags and frozen prior to analysis. In order to extract the neonicotinoids from soil, 5 g of soil was added to a 50-mL centrifuge tube along with 10 μL of a 1-10ng/μL analytical grade standards, 5 mL ddH_2_O, 10mL CAN + 1%HOAC, in addition to 1 g of NaCl and 4 g MgSO_4_. The mixture was hand shaken vigorously for 1 min, and centrifuged at 4,000 rpm for 5 min. Following centrifugation, 1 mL of the supernatant was transferred to a *Quechers* dSPE Tube containing PSA and MgSO_4_, vortexed for 1 min followed by 5 min of centrifugation at 15,000 rpm. The resulting supernatant was transferred to a microcentrifuge tube and dried in a SpeedVac concentrator prior to analysis using QQQ mass spectrometry. Reported analytical results are the mean of three replicate measurements from each sample (see S1 Appendix for concentration determination).

### Ethics statement

The Purdue Institutional Animal Care and Use Committee (IACUC) approved all animal husbandry and euthanasia procedures (protocol #1304000846). Field permits for collecting animals were provided by the Indiana Department of Natural Resources, Division of Fish and Wildlife.

## Results

### LC_50_ tests

**Fig 2:**
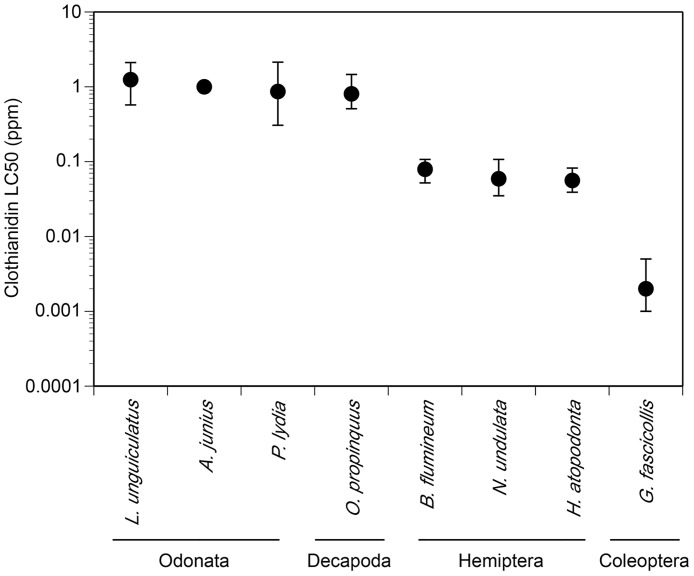
LC_50_ values with 95% confidence intervals for select aquatic macroinvertebrates. The 95% CI for *Anax* could not be calculated because the treatments resulted in 0, 50 or 100% mortality.

Our LC_50_ experiments revealed dramatic differences (several orders of magnitude) in the toxicity of clothianidin to aquatic invertebrates and vertebrates (Table 2, Fig 2). Survival curves are presented in S3 and S4 Figs. In general, predatory invertebrates displayed high sensitivity. Additionally, species within the same order (i.e. Hemiptera, Odonata) tended to cluster in their LC_50_ values. Moreover, the hemipterans were more sensitive than the odonates to clothianidin. The single member of the Coleoptera (*Graphoderus*) had the highest sensitivity to the insecticide. For larvae of the three amphibian species (*L*. *pipiens*, *L*. *clamitans*, and *H*. *versicolor*) and the two snail species (*P*. *acuta*, *H*. *trivolvis*), we were unable to calculate LC_50_ values because there was no mortality at the saturation point of formulated clothianidin (Arena) in water (~327 ppm).

**Table 2:** LC_50_ values and associated 95% confidence intervals for the invertebrate species that experienced mortality when exposed to clothianidin.

Species	LC50_48-h_ (ppm)	95% confidence limit	
		Lower	Upper
*Lestes unguiculatus*	1.245	0.572	2.11
*Anax junius*	1	a	a
*Plathemis lydia*	0.865	0.306	2.133
*Orchonectes propinquus*	0.805	0.509	1.462
*Belostoma flumineum*	0.079	0.052	0.107
*Notonecta undulata*	0.059	0.035	0.107
*Hesperocorixa atopodonta*	0.056	0.039	0.082
*Graphoderus fascicollis*	0.002	0.001	0.005

^a^ = The 95% CI could not be calculated because the treatments resulted in 0, 50 or 100% mortality.

### Sublethal experiments

**Fig 3:**
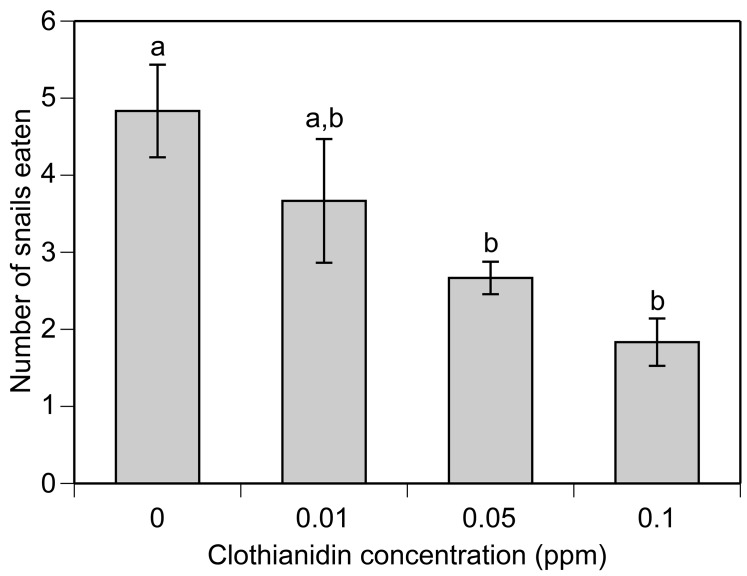
Number of snail prey consumed by water bugs exposed to different clothianidin concentrations. Treatments sharing letters are not significantly different from each other based on pairwise comparisons (Bonferroni corrected P > 0.05). Data are means ± 1 SE.

**Fig 4:**
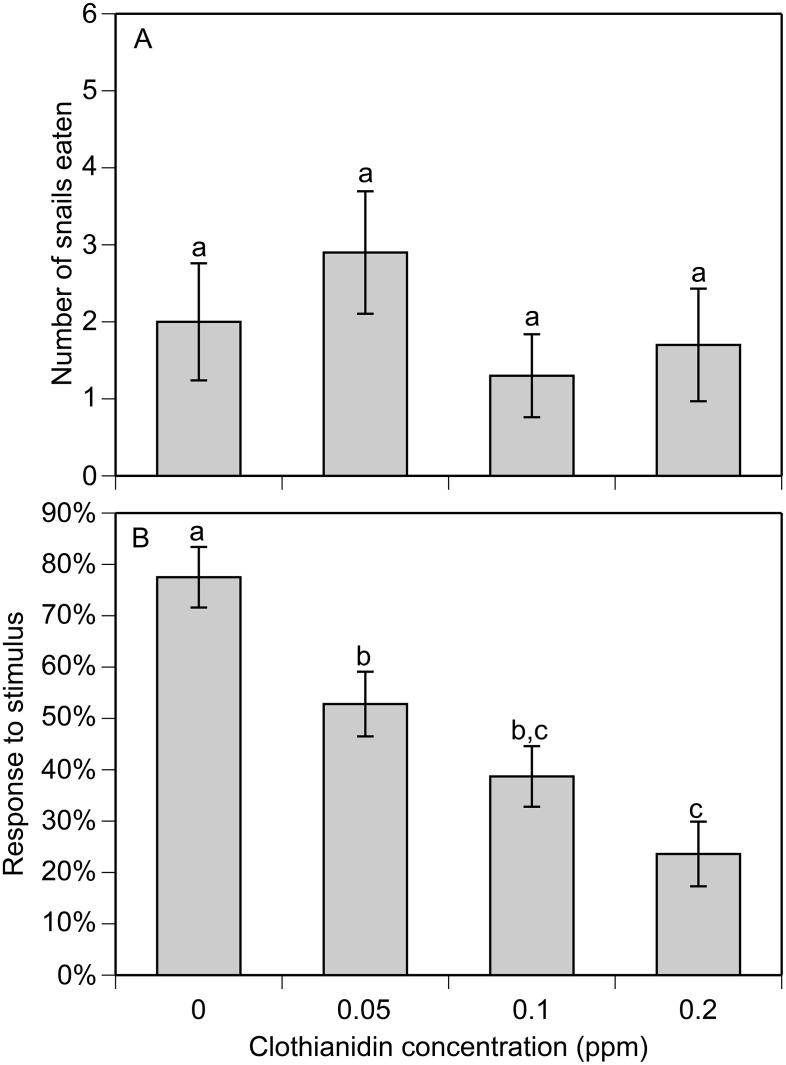
Number of snail prey consumed by crayfish (A) and the percentage of responses to stimulus for crayfish (B). Treatments sharing letters are not significantly different from each other based on pairwise comparisons (Bonferroni corrected P > 0.05). Data are means ± 1 SE.

There was no evidence that clothianidin influenced tadpole behavior (data not shown). While there was a significant effect of elapsed time on tadpole activity (F_3,48_ = 5.6, P = 0.002), there was no effect of clothianidin (F_3,16_ = 1.8, P = 0.197) or time*clothianidin interaction (F_9,48_ = 1.3, P = 0.283). In the predation trials, we found that clothianidin exposure reduced the consumption of prey by water bugs in a dose-dependent manner (F_3,20_ = 5.86, P = 0.005; Fig 3). At the highest clothianidin concentration (0.1 ppm), there was a 62% reduction in prey consumption compared to the control. In contrast, clothianidin exposure did not influence prey consumption in crayfish (F_3,35_ = 0.89, P = 0.445; Fig 4A). However, we did detect a significant dose-dependent effect on their response to stimuli (F_3,34_ = 14.23, P = <0.001; Fig 4B). For example, at the highest clothianidin concentration (0.2 ppm), there was a 70% reduction in stimulus response compared to the control.

### Mesocosm experiment

**Fig 5:**
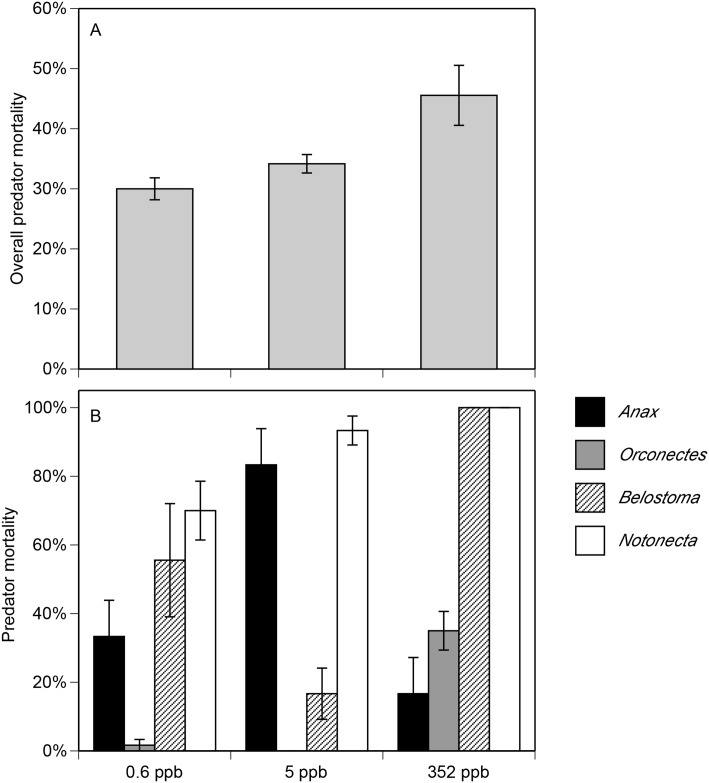
Total predator mortality (A) and the mortality of each predator species (B) following exposure to different clothianidin concentrations. Clothianidin concentrations represent actual concentrations measured in the tanks following addition of Arena granules. Data are means ± 1 SE.

Clothianidin exposure had significant effects on the total mortality of invertebrate predators and the mortality of each species (χ^2^ > 7.8, P ≤ 0.02; S4 Table, Fig 5). Across all predator species, predator mortality increased by 52% with 352 ppb of clothianidin compared to 0.6 ppb (P = 0.011). However, there was no difference between 0.6 ppb and 5 ppb or between 5 ppb and 352 ppb (P ≥ 0.071). When examining the individual predator species, we found that *Notonecta* had high mortality in all treatments. However, there was still a significant increase in mortality in the 352 ppb treatment compared to the 0 ppb treatment (P = 0.005). For *Anax*, mortality was highest at 5 ppb but 50% lower at 0 ppb (P = 0.013) and 80% lower at 352 ppb (P = 0.001), with no differences between 0.6 ppb and 352 ppb (P = 0.843). For *Orconectes* and *Belostoma*, we found no significant difference between 0.6 ppb and 5 ppb (P ≥ 0.056). However, mortality was greater in the 352 ppb treatment compared to the 0.6 ppb and 5 ppb treatments (P ≤ 0.026).

**Fig 6:**
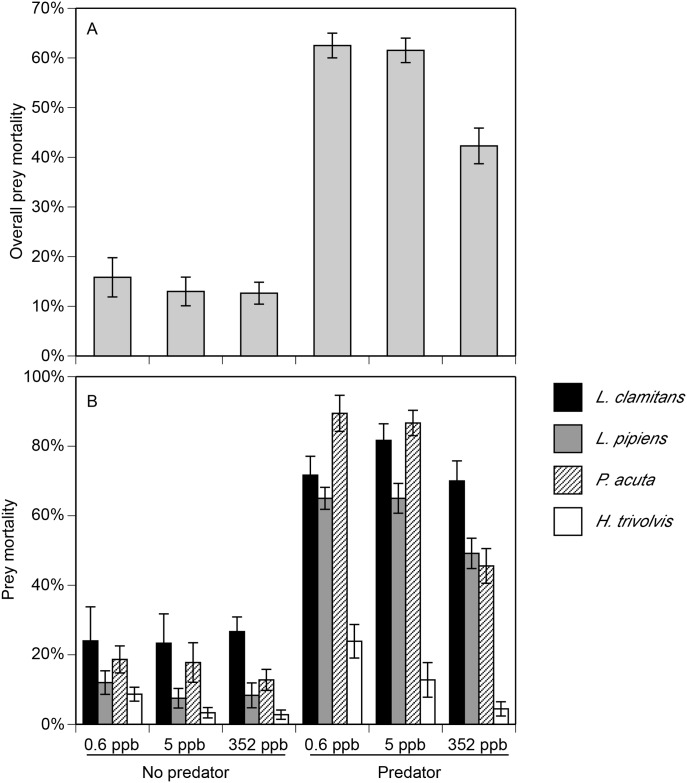
Total prey mortality (A) and the mortality of each predator species (B) following exposure to different clothianidin concentrations and predator environments. Clothianidin concentrations represent actual concentrations measured in the tanks following addition of Arena granules. Data are means ± 1 SE.

There were significant effects of predators, clothianidin, and their interaction on overall prey mortality and the mortality of individual prey species (Fig 6, Table 3). Averaged across the clothianidin treatments, overall prey mortality and the mortality of individual prey species was 9 to 57% higher in the predator treatments compared to the no-predator treatments. In contrast, clothianidin exposure decreased overall prey mortality and the mortality of individual prey species with the exception of *L*. *clamitans*. Averaged across predator treatments, prey mortality was 10 to 25% lower at 352 ppb of clothianidin compared to 0.6 ppb. Lastly, we only observed an interactive effect of predators and clothianidin on overall prey mortality and the mortality of *P*. *acuta*. For both response variables, mortality was relatively low across the clothianidin concentrations in the no-predator treatment. However, mortality in the predator treatment was lower in the 352 ppb treatment compared to the 0.6 ppb and 5 ppb.

**Table 3:** The results of ANOVAs on the mortality of all prey species combined and each individual species when exposed to a factorial combination of predators and clothianidin concentration. Bold P-values are significant at P < 0.05.

Source	d.f.	Prey total		*L*. *pipiens*		*L*. *clamitans*		*P*. *acuta*		*H*. *trivolvis*	
		F	P	F	P	F	P	F	P	F	P
Predator	1,29	299.3	**<0.001**	282.9	**<0.001**	87.5	**<0.001**	238.0	**<0.001**	11.0	**0.002**
Insecticide	2,29	9.2	**<0.001**	3.9	**0.032**	0.3	0.734	18.7	**<0.001**	7.7	**0.002**
Interaction	2,29	6.4	**0.005**	2.8	0.075	0.7	0.494	11.2	**0.001**	2.2	0.131

### Field survey

**Fig 7:**
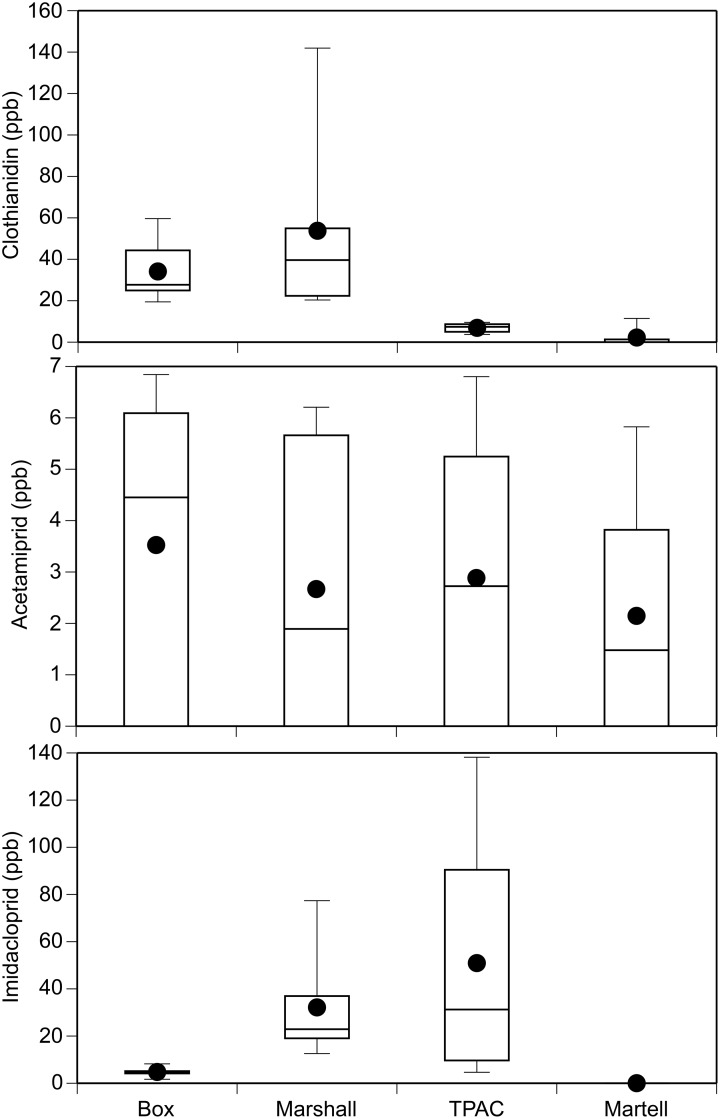
Boxplots of clothianidin, imidacloprid, and acetamiprid concentrations (ppb) detected in soil samples at four sites in Tippecanoe County, Indiana. Data includes samples taken throughout the growing season.

We detected the neonicotinoids acetamiprid, imidacloprid, and clothianidin in 56%, 78%, and 81% of our soil samples, respectively (n = 32 total samples per chemical; Fig 7). The mean concentration of acetamiprid, imidacloprid, and clothianidin across all sites and sampling periods was 2.8, 22.0, and 24.2 ppb, respectively. The maximum concentration of clothianidin, imidacloprid, and acetamiprid across all sites and sample periods was 176, 141, and 7 ppb, respectively. Peak concentrations tended to occur 4 weeks post planting (S5 Table).

**Fig 8:**
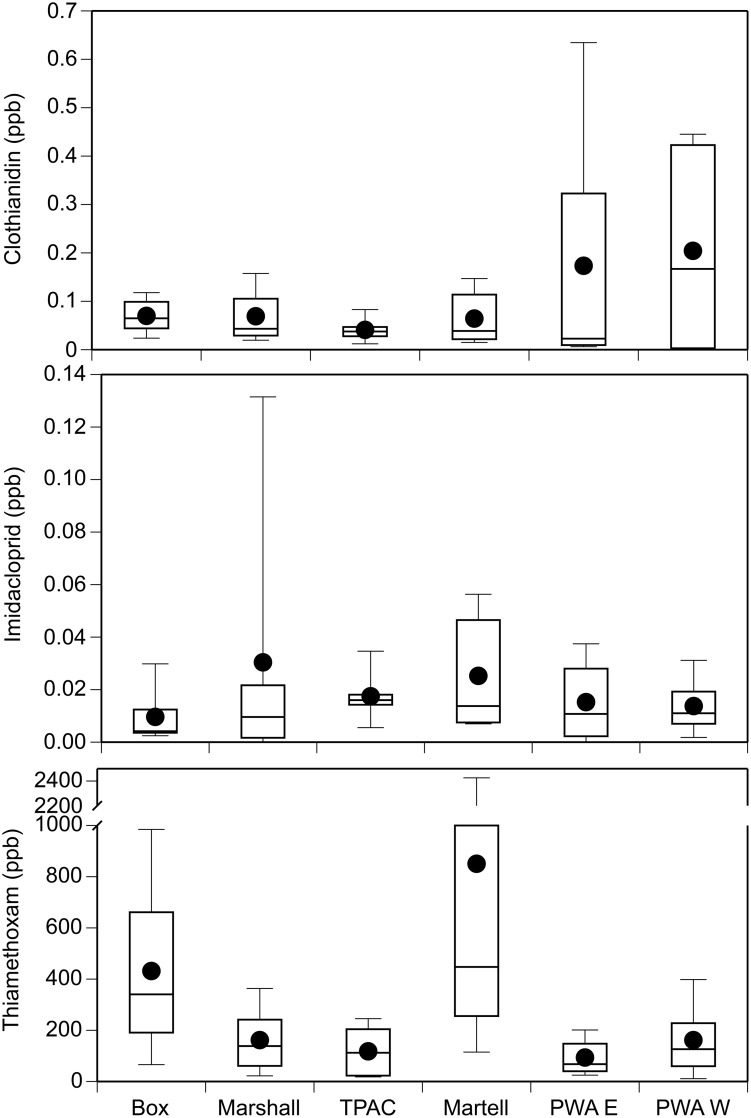
Boxplots of clothianidin, imidacloprid, and thiamethoxam concentrations (ppb) detected in water samples at six sites in Tippecanoe County, Indiana. Data includes samples taken throughout the growing season.

We detected the neonicotinoids clothianidin, imidacloprid, and thiamethoxam in 96%, 90%, and 98% of our water samples, respectively (n = 48 total samples per chemical; Fig 8). The mean concentration of clothianidin, imidacloprid, and thiamethoxam across all sites and sample periods was 0.10, 0.02, and 302 ppb, respectively. The maximum concentration of clothianidin, imidacloprid, and thiamethoxam was 0.67 ppb, 0.18 ppb, and 2,568 ppb, respectively. In general, concentrations tended to peak 5 to 7 weeks post planting (S6 Table).

## Discussion

Neonicotinoids pose a risk to aquatic systems due to their low soil binding, high soil persistence, and high water solubility [45]. Using controlled laboratory experiments, we documented that the neonicotinoid clothianidin has lethal and sublethal effects on wetland invertebrates at field relevant concentrations. Using a community-level mesocosm experiment, we found that clothianidin can reduce the abundance of predatory invertebrates, which indirectly benefits clothianidin-tolerant herbivores in the community. Additionally, we detected four neonicotinoids in the vast majority of soil and water samples at field sites in close proximity to agricultural lands.

Despite the increasing usage of neonicotinoids, toxicity tests with aquatic species have largely focused on the older neonicotinoid imidacloprid [13,46–49]. We found wide variation in the toxicity of clothianidin to the wetland species tested. The most sensitive species was *Graphoderus fascicollis* (Coleoptera) with a LC_50_ value of 0.002 ppm. The current U.S. EPA Aquatic Life Benchmark for clothianidin (acute exposure) and freshwater invertebrates is 0.011 ppm. Yet, clothianidin has been detected in field samples as high as 0.043 ppm [42]. Given that the sensitivity of *G*. *fascicollis* was an order of magnitude lower than the benchmark, future research should consider including species beyond the typical toxicological models (e.g., *Chironomus riparius*, *Mysidopsis bahia*, *Daphnia* spp.) in neonicotinoid risk assessment [50]. For example, the acute toxicity of *Daphnia magna*, a common model for aquatic toxicology, to clothianidin is 67 ppm [37], suggesting that they are remarkably tolerant compared to other invertebrates. Indeed, cladocerans in general tend to display higher tolerance than other aquatic arthropods to neonicotinoids [11,50,51]. We also found that species from the same order displayed similar levels of sensitivity to clothianidin; the odonates had LC_50_ values around 1 ppm while the hemipterans had LC_50_ values around 0.06 ppm. Previous studies have observed phylogenetic relatedness as a predictive factor for toxicity among related species for other contaminants (e.g. endosulfan, zinc, *Bacillus thuringiensis* toxin) [52–54]. Our results provide support for the notion that phylogenetic relatedness may be useful for predicting toxicity of clothianidin and possibly other neonicotinoids in aquatic invertebrates. It is also important to note that several neonicotinoids including clothianidin were detected at our wetland sites, which served as sources for several of our experimental animals. Recent research has demonstrated that non-target aquatic species can evolve tolerance to insecticides (e.g., carbaryl; [55–57]). Thus, our toxicity values could be underestimates of toxicity for populations without a history of neonicotinoid exposure. However, given the widespread neonicotinoid contamination of surface waters in North America [8–10], our results are representative of real-world scenarios.

We also tested two snail species (*H*. *trivolvis* and *P*. *acuta*) and three amphibian species (*H*. *versicolor*, *L*. *pipiens*, *L*. *clamitans*) for their sensitivity to clothianidin. These species displayed high tolerance to the chemical and no individuals died at the highest dissolvable concentration tested (327 ppm). In general, freshwater snails appear to be highly tolerant to a diverse array of insecticides and herbicides [34,58–60]. It was also not surprising that tadpoles were tolerant to clothianidin; neonicotinoids generally have low toxicity in vertebrates [49].

In addition to direct lethal effects, neonicotinoids have been shown to cause a diverse range of sublethal effects on aquatic organisms including effects on feeding, movement, immunity, growth, and development [14–21,48,61]. Using a subset of the species from the LC_50_ tests, we found that sublethal clothianidin concentrations can alter behavior and foraging of predatory invertebrates but not tadpoles. For water bugs, we found that clothianidin reduced the consumption of snails with a ~62% reduction at the highest tested clothianidin concentration (0.1 ppm). In the case of crayfish, we did not observe a similar effect on snail consumption. However, we did observe a reduction in the response to external stimuli (i.e. physical agitation). At the highest concentration of clothianidin (0.2 ppm), crayfish were 70% less responsive compared to the control. For both species, we observed behavioral effects at 0.05 ppm, which is within the range of concentrations detected in water samples taken from agricultural areas [42]. Collectively, these results demonstrate that clothianidin can have sublethal effects on the behavior of aquatic invertebrates, at environmentally relevant concentrations, and provide the basis for future work that investigates potentially important sublethal behavioral effects.

While laboratory experiments documenting the toxicity of neonicotinoids are a critical step in ecotoxicology, there is a need for research that explores the community-level and ecosystem-level consequences of exposure, especially in aquatic systems. Community-level experiments have been conducted with the earliest neonicotinoids, imidacloprid and thiacloprid [15,23,27,62,63], but there is no similar work to report using clothianidin. Thus, we conducted a semi-natural mesocosm experiment to explore the community-level effects of clothianidin exposure. As expected, there was a 52% increase in predator mortality when exposed to 500 ppb clothianidin compared to the control. There was no effect on mortality at 5 ppb. However, there were differences in the response of each predator species to clothianidin. For instance, water bugs and backswimmers were the most heavily affected; 100% mortality occurred in the 352 ppb treatment. In contrast, crayfish displayed much higher tolerance to the insecticide with only 35% mortality at the highest concentration. Dragonfly larvae experienced over 80% mortality at 5 ppb but just 15% mortality at 500 ppb. However, we note that our sample size for dragonfly larvae (n = 2 per tank) was relatively low. Although our experiment included a dose (352 ppb) that was beyond what organisms typically encounter in the field, they collectively reaffirm our predictions regarding the lethal effects of clothianidin at different concentrations, which can be useful in assessing does-response relationships. Lethal effects are admittedly a coarse measurement of insecticide effects, but they provide a foundation for experiments investigating population-level effects upon key sublethal parameters such as growth, feeding and reproduction.

In general, overall prey mortality followed our *a priori* predictions. In the absence of predators, prey mortality was low across clothianidin treatments (between 2% and 25%), which was consistent with our toxicity trials with tadpoles and snails. In treatments containing predators, prey mortality was dependent on the level of clothianidin; there was less prey mortality at 352 ppb clothianidin compared to the control and 5 ppb clothianidin treatments. This indirect effect of clothianidin was likely mediated by a combination of direct lethal effects on the predators and sublethal effects on predator foraging behavior. While water bugs were eliminated from the tanks at 352 ppb, a large proportion of the crayfish and dragonfly larvae remained. Thus, the increase in prey survival at 352 ppb was likely mediated by direct mortality of water bugs and sublethal effects on crayfish and dragonfly larvae foraging. In contrast to the 352 ppb treatment, we did not observe significant changes in prey mortality at 5 ppb. Although dragonfly larvae experienced increased mortality in this treatment, the presence of water bugs and crayfish appeared to compensate for the loss of this predator. Moreover, these results suggest that there were no sublethal effects on predator foraging at 5 ppb. Our data suggest that prey species embedded within communities containing invertebrate predators can benefit from neonicotinoid exposure; these results are not exclusive to neonicotinoids. Ecotoxicology experiments using communities have observed an increase in herbivore survival as a result of predator elimination across a diversity of chemicals including neonicotinoids [21,27,33,48,64–67]. Zooplankton were the only group that were largely unaffected by our treatments. Acute toxicity tests have generally demonstrated that many daphnid, cladoceran, and crustacean species have high tolerance for neonicotinoids [37,50,61]. Moreover, the main zooplankton predator (the backswimmer *N*. *undulata*) exhibited low survival across all treatments, which minimized predator effects on their populations.

Over the course of the 2015 growing season, we monitored water and soil from sites in Tippecanoe County, Indiana that were located near corn and soybean crops to capture the seasonal variation of potential neonicotinoid exposure levels. Clothianidin, imidacloprid, and acetamiprid were detected in soil samples while clothianidin, imidacloprid, and thiamethoxam were detected in water samples. There was broad variation in the detected clothianidin concentration (0 to 176 ppb) in our soil samples. Likewise, the mean and maximum concentration of clothianidin in our water samples was 0.10 ppb and 0.67 ppb, respectively. While imidacloprid has been the focus of most field studies, there are a growing number of studies that have expanded to include clothianidin especially in surface waters [10,13,42,68–71]. Hladik et al. [68] detected levels of clothianidin as high as 0.0257 ppb in the midwestern U.S., and higher concentrations up to 3.1 ppb were found in the prairie pothole region of Canada [10]. However, Schaafsma et al. [42] detected up to 43 ppb of clothianidin in standing water within agricultural fields in Canada. Interestingly, we detected acetamiprid in soil samples but not water samples while the reverse was observed for thiamethoxam. Given that the concentration of acetamiprid in the soil samples was relatively low, it is possible that this insecticide degraded below detectability for our water samples. We detected high concentrations of thiamethoxam in our water samples (mean = 302 ppb, maximum = 2,568 ppb), which is likely due in part to the very high water solubility of this compound [72]. The concentrations we report here are significantly higher than the U.S. EPA Aquatic Life Benchmark (acute exposure) for freshwater invertebrates (17.5 ppb). Interestingly, this insecticide was not detected in our soil samples. For thiamethoxam that is not washed into surface waters, it is possible that soil microorganisms degrade the chemical to its metabolite clothianidin. This may explain the wide range of clothianidin concentrations detected in our soil samples. Moreover, because clothianidin is the toxic metabolite of thiamethoxam, our results suggest that the actual clothianidin concentrations that organisms will encounter is likely to be underestimated by focusing on clothianidin concentrations alone. However, more research is needed to determine the factors contributing to these field concentrations in our study area. Overall, we detected neonicotinoids in >90% of our water samples. Thus, our study adds to the growing evidence that neonicotinoids are ubiquitous contaminants in surface waters [8,11,42,68].

Benthic invertebrates play an important role in energy flow and nutrient cycling in aquatic systems [73]. Consequently, chemical contaminants that enter these systems have the potential to alter community structure and ecosystem function. Our results demonstrate that the neonicotinoid clothianidin can have lethal and sublethal effects on aquatic invertebrates. While more work examining other neonicotinoids is necessary to assess generality, our work combined with existing studies suggest that the most widely used compounds in this insecticide class have the potential to significantly alter aquatic communities, highlighting the need for more research into the community- and ecosystem-level consequences of exposure [74].

## Supporting information

S1 Appendix Supplemental methods and results for the mesocosm experiment. (PDF)

S1 Fig Phytoplankton measurements for the two sampling periods (days 11 and 18 of the experiment). (PDF)

S2 Fig pH measurements for the two sampling periods (days 11 and 18 of the experiment). (PDF)

S3 Fig Survival curves for (A) *Lestes unguiculatus*, (B) *Anax junius*, (C) *Plathemis lydia*, and (D) *Orconectes propinquus* in the 48 hr LC_50_ tests. (PDF)

S4 Fig Survival curves for (A) *Belostoma flumineum*, (B) *Notonecta undulata*, (C) *Hesperocorixa atopodonta*, and (D) *Graphoderus fascicollis* in the 48 hr LC_50_ tests. (PDF)

S1 Table QQQ mass spectrometry measurements of clothianidin over time in the three insecticide treatments from the mesocosm experiment. (PDF)

S2 Table Results of repeated-measures MANOVA on the effects of predators and clothianidin concentration on periphyton, phytoplankton, and zooplankton on the two sample dates. (PDF)

S3 Table Results of repeated-measures MANOVA on the effects of predators and clothianidin concentration on temperature, conductivity, and pH on the two sample dates. (PDF)

S4 Table The results of analyses on the survival and biomass of all predator species combined and each individual species when exposed to different levels of clothianidin. (PDF)

S5 Table Mean concentrations (ppb) of neonicotinoids detected in soil samples at four sites in Tippecanoe County, IN over the 2015 planting season. (PDF)

S6 Table Mean concentrations (ppb) of neonicotinoids detected in water samples at six sites in Tippecanoe County, IN over the 2015 planting season. (PDF)
