# Correction: Effects of clothianidin on aquatic communities: Evaluating the impacts of lethal and sublethal exposure to neonicotinoids

**DOI:** 10.1371/journal.pone.0194634

**Published:** 2018-03-15

**Authors:** Jesse C. Miles, Jessica Hua, Maria S. Sepulveda, Christian H. Krupke, Jason T. Hoverman

The authors report a substantial error in one of the datasets. This error involved the reported values for the insecticide thiamethoxam in water samples from the field study.

The errors within the text are listed below along with the corrected figure and SI file: Fig 8 and S6 Table.

There are errors in the penultimate two sentences of the Abstract. The correct sentences are: In water samples, we detected clothianidin (max = 0.67 ppb), imidacloprid (max = 0.18 ppb), and thiamethoxam (max = 0.02 ppb). Neonicotinoids were detected in >56% of soil samples and >87% of the water samples, which reflects a growing understanding that neonicotinoids are ubiquitous environmental contaminants.

There are errors in the first three sentences of the final paragraph of the Results section. The correct sentences are: We detected the neonicotinoids clothianidin, imidacloprid, and thiamethoxam in 96%, 90%, and 77% of our water samples, respectively (n = 48 total samples per chemical; Fig 8). The mean concentration of clothianidin, imidacloprid, and thiamethoxam across all sites and sample periods was 0.10, 0.02, and 0.003 ppb, respectively. The maximum concentration of clothianidin, imidacloprid, and thiamethoxam was 0.67 ppb, 0.18 ppb, and 0.02 ppb, respectively.

There are errors in the penultimate paragraph of the Discussion section. Sentences 10–15 are removed and the penultimate sentence is amended. The correct paragraph is: Over the course of the 2015 growing season, we monitored water and soil from sites in Tippecanoe County, Indiana that were located near corn and soybean crops to capture the seasonal variation of potential neonicotinoid exposure levels. Clothianidin, imidacloprid, and acetamiprid were detected in soil samples while clothianidin, imidacloprid, and thiamethoxam were detected in water samples. There was broad variation in the detected clothianidin concentration (0 to 176 ppb) in our soil samples. Likewise, the mean and maximum concentration of clothianidin in our water samples was 0.10 ppb and 0.67 ppb, respectively. While imidacloprid has been the focus of most field studies, there are a growing number of studies that have expanded to include clothianidin especially in surface waters [10,13,42,68–71]. Hladik et al. [68] detected levels of clothianidin as high as 0.0257 ppb in the midwestern U.S., and higher concentrations up to 3.1 ppb were found in the prairie pothole region of Canada [10]. However, Schaafsma et al. [42] detected up to 43 ppb of clothianidin in standing water within agricultural fields in Canada. Interestingly, we detected acetamiprid in soil samples but not water samples while the reverse was observed for thiamethoxam. Given that the concentration of acetamiprid in the soil samples was relatively low, it is possible that this insecticide degraded below detectability for our water samples. Overall, we detected neonicotinoids in >87% of our water samples. Thus, our study adds to the growing evidence that neonicotinoids are ubiquitous contaminants in surface waters [8,11,42,68].

Fig 8 is incorrect. The authors have provided a corrected version here.

**Fig 8:**
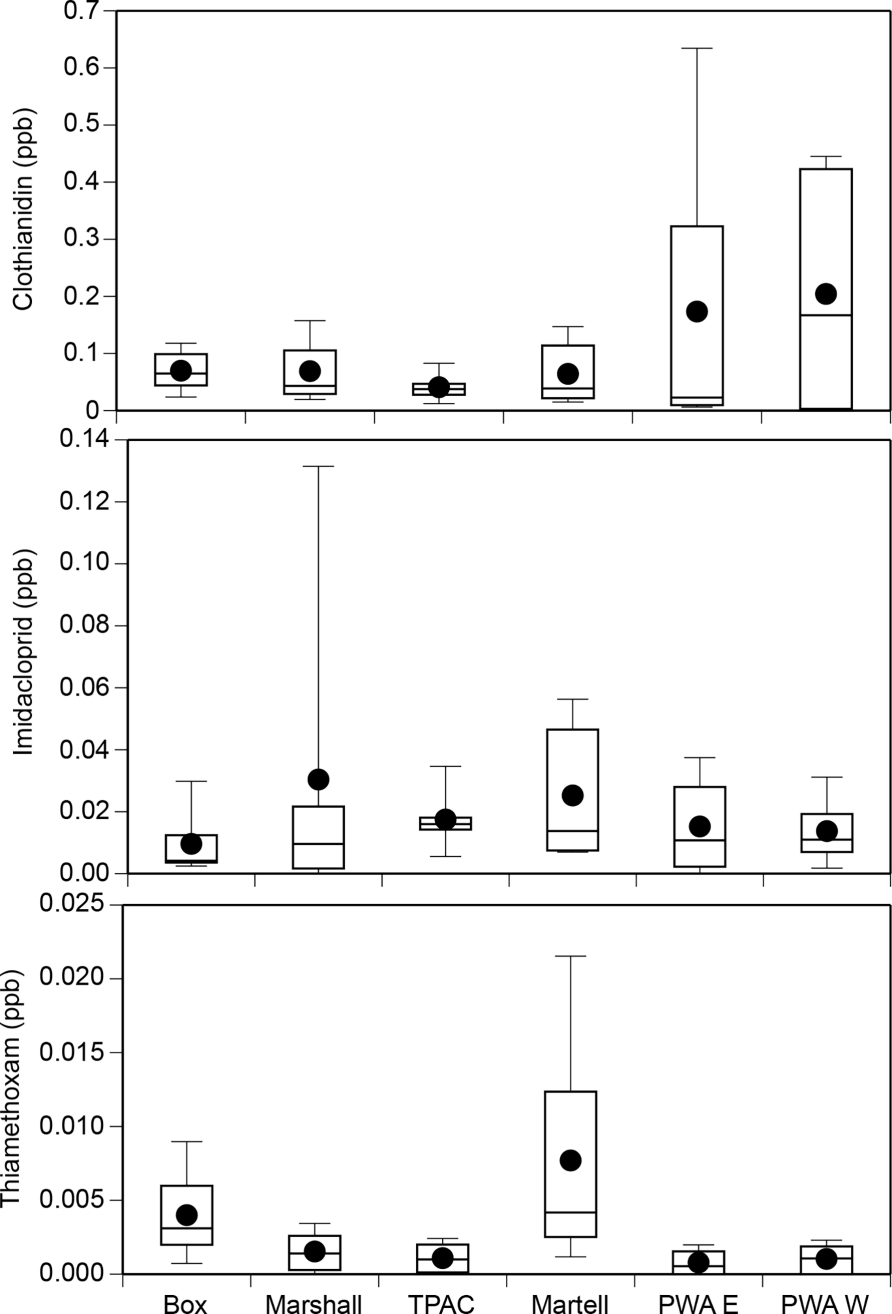
Boxplots of clothianidin, imidacloprid, and thiamethoxam concentrations (ppb) detected in water samples at six sites in Tippecanoe County, Indiana. Data includes samples taken throughout the growing season.

S6 Table is incorrect. The authors have provided a corrected version here.

## Supporting information

S6 Table Mean concentrations (ppb) of neonicotinoids detected in water samples at six sites in Tippecanoe County, IN over the 2015 planting season. (PDF)
