# Supplementary material for: Effects of clothianidin on aquatic communities: Evaluating the impacts of lethal and sublethal exposure to neonicotinoids
Source: PLoS One. 2017 Mar 23;12(3):e0174171. doi: 10.1371/journal.pone.0174171 (PMC5363855; doi:10.1371/journal.pone.0174171)
Supplement: S1 Appendix — (PDF) [file pone.0174171.s001.pdf]

S1 Appendix. Supplemental methods and results for the mesocosm experiment

We measured pH, temperature, and conductivity on days 11 and 18 July using a YSI Professional Plus probe. The probe was suspended in a central location in the water column during measurement. Phytoplankton was measured on days 11 and 18 in each tank by pooling 300 mL of water from three different locations throughout the tank. The locations were standardized across the tanks. The samples were then filtered through Whatman GF/C filters (90 mm) and stored in a freezer until chlorophyll A extraction. The GF/C filters were first cut into small pieces and placed into vials of 90% acetone. A sonicator was then used to further homogenize the mixture and 3 mL of the extract was placed in a quartz cuvette. A NanoDrop spectrophotometer was used to measure absorbance at 664 nm and 750 nm (turbidity correction). The extract was then acidified using 0.1N HCl and measured at 665 (pheophyton correction). The corrected chlorophyll a concentration (ug L^{-1}) was determined using the following formula:

Chlorophyll *a* corrected (ug/L)= $[26.7(664\text{-}750) - 665A) \times V1] / (V2 \times L)$

where:

665A = turbidity-corrected absorbance at 665 nm after acidification

V1 = volume of extractant (mL)

V2 = volume of sample filtered (L)

L = path length (cm)

Periphyton biomass was measured on days 11 and 18. To measure periphyton biomass, the clay tile was brought indoors and placed into a 10-L tub containing a small amount of water. The tile was scrubbed using a toothbrush to remove attached algae. The water was then filtered through a pre-weighed Whatman GF/C filter (90 mm) that had been dried for 24 hrs at 60°C. The filters were dried for 24 hrs at 60°C and weighed to determine periphyton biomass.

We sampled zooplankton populations on days 11 and 18. Samples were collected from five locations in the tank (each cardinal direction and the center of the tank) using a 150 mm PVC pipe capped at each end with a tennis ball. Water was then filtered through Nitex (67 Micron) screening and zooplankton stored in 70% ethanol in vials for quantification. We processed the samples by inverting each vial several times and removing five 5-mL subsamples. For each subsample, the number of zooplankton was enumerated. Following enumeration, total volume of ethanol was measured for the vial. We then calculated the average abundance per mL of the five samples and extrapolated this value to the total volume of the vial.

Because we sampled zooplankton, phytoplankton, periphyton, and abiotics (temperature, conductivity, and pH) twice during the experiment, we conducted repeated-measures multivariate analysis of variance (rm-MANOVA). We conducted one rmMANOVA using zooplankton, phytoplankton, and periphyton and a second with the abiotic data. In the analyses, we examined the effects of time, predators, clothianidin, and their interactions. For significant univariate effects, we conducted mean comparisons using Bonferroni correction.

Results: Mesocosm experiment

We used rm-MANOVA to assess the effects of time and our treatments on zooplankton abundance, phytoplankton abundance (chl A), and periphyton biomass. We found significant multivariate effects of time, clothianidin concentration, predator*clothianidin interaction, and time*predator*clothianidin interaction (S3 Table). In examining each response variable, we found no time or treatment effects for zooplankton. For periphyton, we found a significant effect of time. Averaged across the treatments, periphyton biomass was 50% lower on day 11 compared to day 18. Phytoplankton abundance was the main driver of the multivariate effects. We found significant effects of time, clothianidin, predator*clothianidin interaction, and

time*predator*clothianidin interaction (S3 Table, S1 Figure). To examine the source of this interaction, we conducted analyses within each sample period. On day 11, there was no effect of predators ($F_{1,29} = 2.9$, $P = 0.099$), clothianidin concentration ($F_{2,29} = 3.2$, $P = 0.054$), or predator*clothianidin interaction ($F_{2,29} = 0.6$, $P = 0.561$). On day 18, there was a significant effect of predators ($F_{1,29} = 14.0$, $P < 0.001$), clothianidin concentration ($F_{2,29} = 13.8$, $P < 0.001$), and predator*clothianidin interaction ($F_{2,29} = 15.7$, $P < 0.001$). The interaction was driven by the 352 ppb treatment within the predator treatment. Phytoplankton abundance was 3 to 28X greater in this treatment compared to all the other treatments ($P < 0.001$). However, there were no differences among the remaining treatments ($P \geq 0.118$).

We used rm-MANOVA to assess the effects of time and our treatments on temperature, conductivity, and pH. We found significant multivariate effects of time, predators, time*clothianidin interaction, and predator*clothianidin interaction (S4 Table). In examining each response variable, we found a significant effect of time for temperature. Averaged across the treatments, temperature was 5% lower on day 11 compared to day 18. For conductivity, we found a significant effect of time and the predator*clothianidin interaction. Averaged across the treatments, conductivity was <1% higher on day 11 compared to day 18. The predator*clothianidin interaction was largely driven by a single treatment. Conductivity in no-predator, 0.6 ppb treatment was 4 to 5% higher compared to the other two no-predator treatments and the predator, 0.6 ppb treatment ($P \leq 0.039$). There were no other differences among treatments ($P \geq 0.134$). The main driver of the multivariate effects was pH. We found significant effects of time, predators, time*clothianidin interaction, predator*clothianidin interaction, and the time*predator*clothianidin interaction (S2 Figure, S4 Table). To examine the source of the interactions, we conducted analyses within each sample period. On both dates,

there was a significant effect of predators ($F_{1,29} \geq 5.4$, $P \leq 0.027$), clothianidin concentration ($F_{2,29} \geq 6.9$, $P \leq 0.004$), and predator*clothianidin interaction ($F_{2,29} \geq 4.9$, $P \leq 0.014$). On day 11 within each predator treatment, pH tended to be higher in the 352 ppb treatment compared to the other two clothianidin concentrations. On day 18 within each predator treatment, pH tended to be higher in the 0.6 and/or 5 ppb treatment compared to the 352 ppb treatment. Given that the maximum different between any of the treatments within the sample dates was less than 2%, we will not address these treatment differences in detail.

Concentration determination

An Agilent 1200 Rapid Resolution LC system coupled to an Agilent 6460 series QQQ MS was used to analyze pesticides in our stock solution, experimental and environmental samples. Insecticide concentrations were determined using a modified QuEChERS protocol [1]. An Agilent Zorbax SB-Phenyl 4.6 mm x 150 mm, 5 μm column was used for LC separation (Agilent Technologies, Santa Clara, CA). The buffers were (A) water + 5 mM ammonium acetate + 0.1 % formic acid and (B) acetonitrile (90%) + 5 mM ammonium acetate (10%) + 0.1% formic acid. The linear LC gradient was as follows: time 0 min, 20% B; time 0.5 min, 20% B; time 10 min, 100% B; time 11 min, 100% B; time 11.5 min, 20% B; time 15 min, 20% B. The flow rate was 0.8 mL/min. Multiple reaction monitoring was used for MS analysis. The data were acquired in positive or negative electrospray ionization (ESI) mode. The jet stream ESI interface had a gas temperature of 330°C, gas flow rate of 10 L/min, nebulizer pressure of 35 psi, sheath gas temperature of 250°C, sheath gas flow rate of 7 L/min, capillary voltage of 4000 V in positive mode/3500 V in negative mode, and nozzle voltage of 1000 V. The ΔEMV voltage was 300 in both positive and negative modes.

Literature Cited

1. Dankyi E, Carboo D, Gordon C, Fomsgaard IS. Application of the QuEChERS procedure and LC-MS/MS for the assessment of neonicotinoid insecticide residues in cocoa beans and shells. J Food Compos Anal. 2015. 44:149-57.
