# Supplementary material for: Effects of clothianidin on aquatic communities: Evaluating the impacts of lethal and sublethal exposure to neonicotinoids
Source: PLoS One. 2017 Mar 23;12(3):e0174171. doi: 10.1371/journal.pone.0174171 (PMC5363855; doi:10.1371/journal.pone.0174171)
Supplement: S1 Fig — (PDF) [file pone.0174171.s002.pdf]

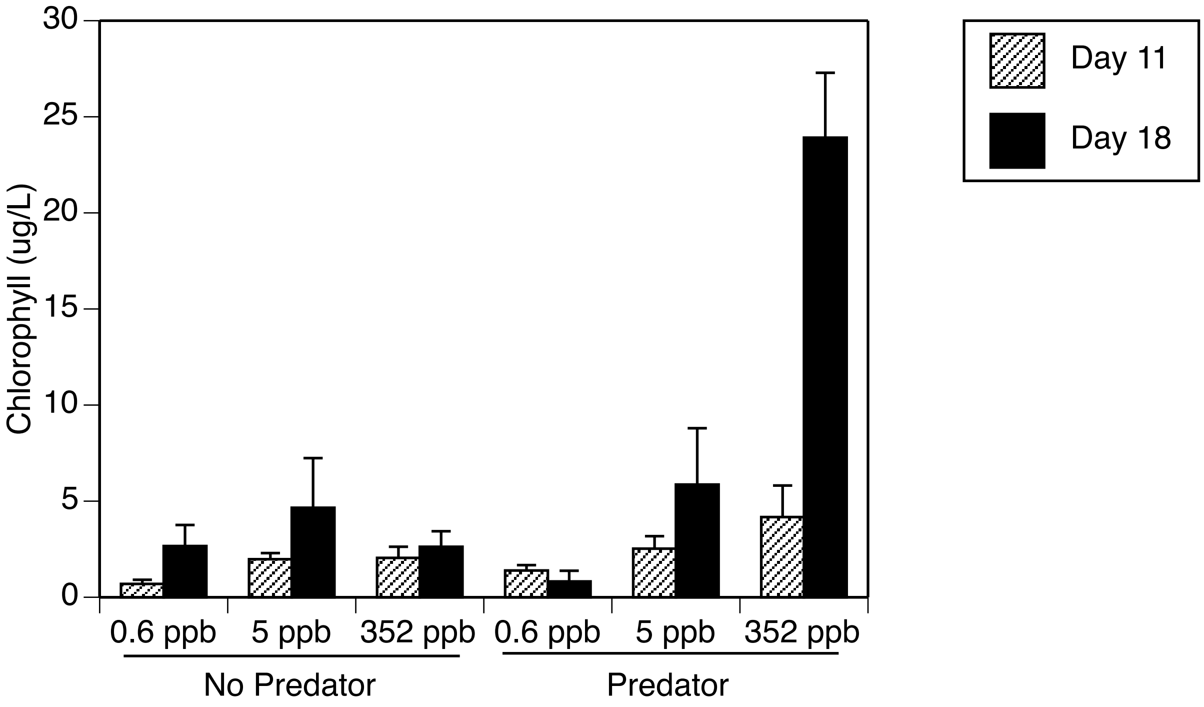

S1 Figure. Phytoplankton measurements for the two sampling periods (days 11 and 18 of the experiment). Time periods are represented by patterns denoted in the figure legend. Data are means + 1 SE.
