# Supplementary material for: Effects of clothianidin on aquatic communities: Evaluating the impacts of lethal and sublethal exposure to neonicotinoids
Source: PLoS One. 2017 Mar 23;12(3):e0174171. doi: 10.1371/journal.pone.0174171 (PMC5363855; doi:10.1371/journal.pone.0174171)
Supplement: S2 Fig — (PDF) [file pone.0174171.s003.pdf]

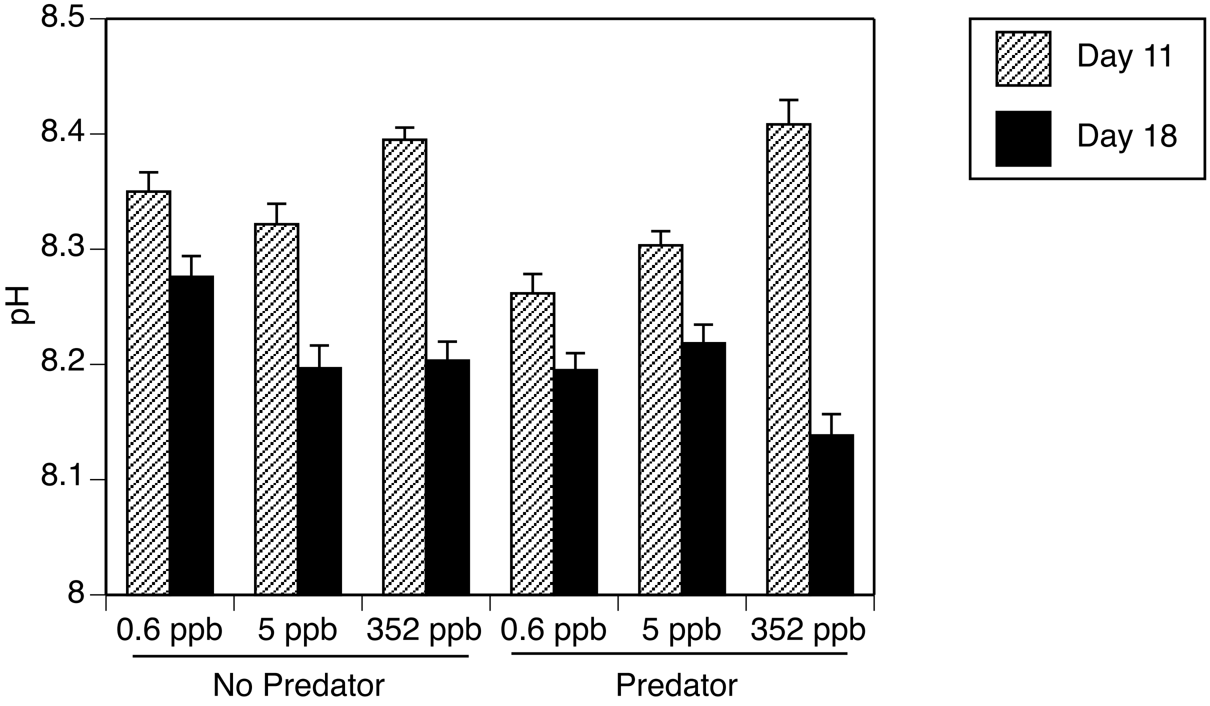

S2 Figure. pH measurements for the two sampling periods (days 11 and 18 of the experiment). Time periods are represented by patterns denoted in the figure legend. Data are means + 1 SE.
