# Supplementary material for: Effects of clothianidin on aquatic communities: Evaluating the impacts of lethal and sublethal exposure to neonicotinoids
Source: PLoS One. 2017 Mar 23;12(3):e0174171. doi: 10.1371/journal.pone.0174171 (PMC5363855; doi:10.1371/journal.pone.0174171)
Supplement: S4 Fig — (PDF) [file pone.0174171.s005.pdf]

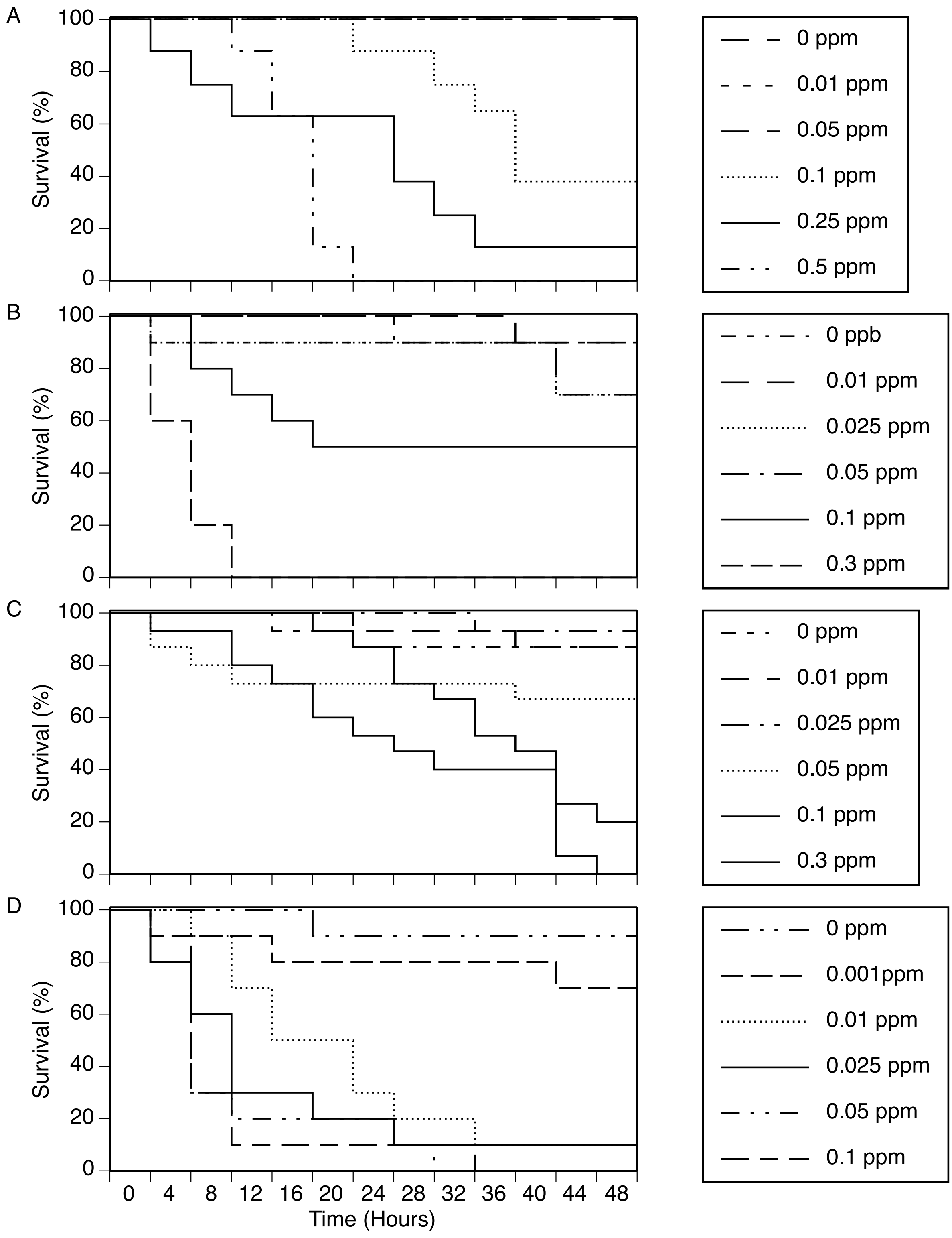

S4 Figure. Survival curves for (A) *Belostoma flumineum*, (B) *Notonecta undulata*, (C) *Hesperocorixa atopodonta*, and (D) *Graphoderus fascicollis* in the 48 hr LC_{50} tests.
