# Supplementary material for: Effects of clothianidin on aquatic communities: Evaluating the impacts of lethal and sublethal exposure to neonicotinoids
Source: PLoS One. 2017 Mar 23;12(3):e0174171. doi: 10.1371/journal.pone.0174171 (PMC5363855; doi:10.1371/journal.pone.0174171)
Supplement: S1 Table — (PDF) [file pone.0174171.s006.pdf]

**S1 Table. QQQ mass spectrometry measurements of clothianidin over time in the three insecticide treatments from the mesocosm experiment.** Measurements are in parts per billion (ppb).

| Treatment | Time | Concentration (ppb) |
|---|---|---|
| Control | Day 0 | 0.657 |
| Control | Day 2 | 0.847 |
| Control | Day 21 | 0.289 |
| Low (10 ppb) | Day 0 | 5.305 |
| Low (10 ppb) | Day 1 | 5.313 |
| Low (10 ppb) | Day 2 | 4.007 |
| Low (10 ppb) | Day 21 | 1.532 |
| High (500 ppb) | Day 0 | 351.938 |
| High (500 ppb) | Day 1 | 199.283 |
| High (500 ppb) | Day 2 | 199.067 |
| High (500 ppb) | Day 21 | 77.550 |
