# Supplementary material for: Effects of clothianidin on aquatic communities: Evaluating the impacts of lethal and sublethal exposure to neonicotinoids
Source: PLoS One. 2017 Mar 23;12(3):e0174171. doi: 10.1371/journal.pone.0174171 (PMC5363855; doi:10.1371/journal.pone.0174171)
Supplement: S2 Table — (PDF) [file pone.0174171.s007.pdf]

S2 Table. Results of repeated-measures MANOVA on the effects of predators and clothianidin concentration on periphyton, phytoplankton, and zooplankton on the two sample dates. Bold P-values are significant at P <0.05.

		Multivariate test			Univariate tests (*P* values)		
		df	F	P	Periphyton	Phytoplankton	Zooplankton
Within subjects	Time	3,27	10.5	**<0.001**	**<0.001**	**<0.001**	0.218
	Time*Predator	3,27	1.3	0.300	0.490	0.424	0.169
	Time*Clothianidin	6,54	1.5	0.195	0.980	**0.036**	0.249
	Time* Predator*Clothianidin	6,54	3.3	**0.007**	**0.063**	**<0.001**	0.867
Between subjects	Predator	3,27	2.4	0.093	0.143	**0.016**	0.954
	Clothianidin	6,54	3.6	**0.004**	0.990	**<0.001**	0.922
	Predator*Clothianidin	6,54	3.3	**0.008**	0.181	**0.002**	0.497
