# Supplementary material for: Effects of clothianidin on aquatic communities: Evaluating the impacts of lethal and sublethal exposure to neonicotinoids
Source: PLoS One. 2017 Mar 23;12(3):e0174171. doi: 10.1371/journal.pone.0174171 (PMC5363855; doi:10.1371/journal.pone.0174171)
Supplement: S3 Table — (PDF) [file pone.0174171.s008.pdf]

Table S3. Results of repeated-measures MANOVA on the effects of predators and clothianidin concentration on temperature, conductivity, and pH on the two sample dates. Bold P-values are significant at P <0.05.

		Multivariate test[1]			Univariate tests (*P* values)		
		df	F	P	Temperature	Conductivity	pH
Within subjects	Time	3,27	753.5	**<0.001**	**<0.001**	**0.013**	**<0.001**
	Time*Predator	3,27	1.7	0.184	**0.036**	0.540	0.591
	Time*Clothianidin	6,54	6.6	**<0.001**	0.194	0.151	**<0.001**
	Time* Predator*Clothianidin	6,54	1.6	0.166	0.288	0.841	**0.042**
Between subjects	Predator	3,27	5.6	**0.004**	0.846	0.672	**0.001**
	Clothianidin	6,54	1.3	0.295	0.123	0.460	0.108
	Predator*Clothianidin	6,54	2.5	**0.033**	0.640	**0.038**	**0.005**
