# Supplementary material for: Effects of clothianidin on aquatic communities: Evaluating the impacts of lethal and sublethal exposure to neonicotinoids
Source: PLoS One. 2017 Mar 23;12(3):e0174171. doi: 10.1371/journal.pone.0174171 (PMC5363855; doi:10.1371/journal.pone.0174171)
Supplement: S4 Table — (PDF) [file pone.0174171.s009.pdf]

S4 Table. The results of analyses on the survival and biomass of all predator species combined and each individual species when exposed to different levels of clothianidin. P-values are listed with associated Chi-squared-statistics; bold P-values are significant at $P < 0.05$.

		Mortality		Mass	
Source	d.f	Chi-squared	P	Chi-squared	P
Total	2	7.85	**0.020**	10.72	**0.005**
Anax	2	9.77	**0.008**	5.73	0.057
Orconectes	2	30.62	**<0.001**	6.94	**0.030**
Belostoma	2	13.99	**<0.001**	7.6	**0.022**
Notonectid	2	9.62	**0.009**	5.63	0.060
