# Supplementary material for: Effects of clothianidin on aquatic communities: Evaluating the impacts of lethal and sublethal exposure to neonicotinoids
Source: PLoS One. 2017 Mar 23;12(3):e0174171. doi: 10.1371/journal.pone.0174171 (PMC5363855; doi:10.1371/journal.pone.0174171)
Supplement: S5 Table — (PDF) [file pone.0174171.s010.pdf]

S5 Table. Mean concentrations (ppb) of neonicotinoids detected in soil samples at four sites in Tippecanoe County, IN over the 2015 planting season

			Week post-planting						
**Chemical**	**Site**	**Pre-planting**	**2**	**3**	**4**	**5**	**6**	**7**	**8**
Clothianidin	Box	27.911	27.432	60.948	32.042	56.573	27.488	22.461	18.159
	Marshall	23.193	36.887	19.852	176.471	48.549	42.342	21.494	61.327
	TPAC	8.565	9.887	8.784	5.054	8.008	4.920	3.232	6.778
	Martell	0.000	0.000	15.237	0.000	2.644	0.000	0.000	0.000
Acetamiprid	Box	5.978	7.116	6.204	4.329	4.575	0.000	0.000	0.000
	Marshall	6.122	6.242	5.197	3.785	0.000	0.000	0.000	0.000
	TPAC	0.000	7.124	4.445	6.048	2.806	2.643	0.000	0.000
	Martell	0.000	6.573	3.561	4.081	2.957	0.000	0.000	0.000
Imidacloprid	Box	3.765	4.702	9.412	4.751	4.539	5.424	4.849	0.799
	Marshall	10.414	50.268	20.444	23.633	88.989	22.303	23.495	17.627
	TPAC	7.507	47.982	141.283	50.019	130.899	3.409	11.806	14.491
	Martell	0.000	0.000	0.000	0.000	0.000	0.000	0.000	0.202
