# Supplementary material for: Effects of clothianidin on aquatic communities: Evaluating the impacts of lethal and sublethal exposure to neonicotinoids
Source: PLoS One. 2017 Mar 23;12(3):e0174171. doi: 10.1371/journal.pone.0174171 (PMC5363855; doi:10.1371/journal.pone.0174171)
Supplement: S6 Table — (PDF) [file pone.0174171.s011.pdf]

S6 Table. Mean concentrations (ppb) of neonicotinoids detected in water samples at six sites in Tippecanoe County, IN over the 2015 planting season.

			Week post-planting						
**Chemical**	**Site**	**Pre-planting**	**2**	**3**	**4**	**5**	**6**	**7**	**8**
Clothianidin	Box	0.103	0.057	0.019	0.054	0.034	0.124	0.094	0.072
	Marshall	0.038	0.039	0.047	0.019	0.020	0.178	0.101	0.109
	TPAC	0.006	0.043	0.027	0.032	0.044	0.050	0.028	0.097
	Martell	0.018	0.025	0.040	0.014	0.037	0.153	0.134	0.094
	PWA E	0.034	0.006	0.008	0.012	0.097	0.012	0.548	0.671
	PWA W	0.000	0.409	0.267	0.067	0.006	0.436	0.000	0.449
Imidacloprid	Box	0.002	0.004	0.004	0.035	0.004	0.019	0.006	0.004
	Marshall	0.000	0.019	0.177	0.003	0.000	0.025	0.008	0.011
	TPAC	0.002	0.016	0.014	0.016	0.017	0.019	0.014	0.041
	Martell	0.007	0.008	0.015	0.007	0.012	0.049	0.045	0.060
	PWA E	0.025	0.000	0.000	0.009	0.013	0.005	0.032	0.040
	PWA W	0.006	0.012	0.018	0.008	0.011	0.021	0.000	0.036
Thiamethoxam	Box	339.791	275.822	48.703	341.091	106.071	903.767	1019.175	418.612
	Marshall	84.303	105.508	285.535	37.582	15.426	396.668	171.397	197.644
	TPAC	22.380	23.405	16.114	120.412	248.222	238.994	169.560	104.156
	Martell	312.631	287.166	224.167	67.969	582.359	2568.050	2093.764	668.123
	PWA E	43.475	134.855	19.358	42.535	37.545	92.036	160.523	218.741
	PWA W	0.000	155.602	241.269	36.961	214.435	82.503	465.630	96.918
