# Supplementary material for: Correction: Effects of clothianidin on aquatic communities: Evaluating the impacts of lethal and sublethal exposure to neonicotinoids
Source: PLoS One. 2018 Mar 15;13(3):e0194634. doi: 10.1371/journal.pone.0194634 (PMC5854427; doi:10.1371/journal.pone.0194634)
Supplement: S6 Table — (PDF) [file pone.0194634.s001.pdf]

S6 Table. Mean concentrations (ppb) of neonicotinoids detected in water samples at six sites in Tippecanoe County, IN over the 2015 planting season.

			Week post-planting						
**Chemical**	**Site**	**Pre-planting**	**2**	**3**	**4**	**5**	**6**	**7**	**8**
Clothianidin	Box	0.103	0.057	0.019	0.054	0.034	0.124	0.094	0.072
	Marshall	0.038	0.039	0.047	0.019	0.020	0.178	0.101	0.109
	TPAC	0.006	0.043	0.027	0.032	0.044	0.050	0.028	0.097
	Martell	0.018	0.025	0.040	0.014	0.037	0.153	0.134	0.094
	PWA E	0.034	0.006	0.008	0.012	0.097	0.012	0.548	0.671
	PWA W	0.000	0.409	0.267	0.067	0.006	0.436	0.000	0.449
Imidacloprid	Box	0.002	0.004	0.004	0.035	0.004	0.019	0.006	0.004
	Marshall	0.000	0.019	0.177	0.003	0.000	0.025	0.008	0.011
	TPAC	0.002	0.016	0.014	0.016	0.017	0.019	0.014	0.041
	Martell	0.007	0.008	0.015	0.007	0.012	0.049	0.045	0.060
	PWA E	0.025	0.000	0.000	0.009	0.013	0.005	0.032	0.040
	PWA W	0.006	0.012	0.018	0.008	0.011	0.021	0.000	0.036
Thiamethoxam	Box	0.003	0.003	0.001	0.003	0.001	0.008	0.009	0.004
	Marshall	0.001	0.001	0.003	0.000	0.000	0.004	0.002	0.002
	TPAC	0.000	0.000	0.000	0.002	0.002	0.002	0.002	0.000
	Martell	0.003	0.003	0.002	0.001	0.005	0.023	0.019	0.006
	PWA E	0.000	0.001	0.000	0.000	0.000	0.001	0.002	0.002
	PWA W	0.000	0.002	0.002	0.000	0.002	0.001	0.000	0.001
